# Music and Emotions in the Brain: Familiarity Matters

**DOI:** 10.1371/journal.pone.0027241

**Published:** 2011-11-16

**Authors:** Carlos Silva Pereira, João Teixeira, Patrícia Figueiredo, João Xavier, São Luís Castro, Elvira Brattico

**Affiliations:** 1 Institute for Biomedical Sciences Abel Salazar (ICBAS), University of Porto, Porto, Portugal; 2 Neuroradiology Department, Hospital Geral de Santo António, Porto, Portugal; 3 Institute of Systems and Robotics, Lisbon & Bioengineering Department, Instituto Superior Técnico, Lisbon, Portugal; 4 Faculty of Psychology and Educational Sciences, University of Porto, Porto, Portugal; 5 Cognitive Brain Research Unit, Institute of Behavioural Sciences, University of Helsinki, Helsinki, Finland; 6 Centre of Excellence in Interdisciplinary Music Research, University of Jyväskylä, Jyväskylä, Finland; John Hopkins School of Medicine, United States of America

## Abstract

The importance of music in our daily life has given rise to an increased number of studies addressing the brain regions involved in its appreciation. Some of these studies controlled only for the familiarity of the stimuli, while others relied on pleasantness ratings, and others still on musical preferences. With a listening test and a functional magnetic resonance imaging (fMRI) experiment, we wished to clarify the role of familiarity in the brain correlates of music appreciation by controlling, in the same study, for both familiarity and musical preferences. First, we conducted a listening test, in which participants rated the familiarity and liking of song excerpts from the pop/rock repertoire, allowing us to select a personalized set of stimuli per subject. Then, we used a passive listening paradigm in fMRI to study music appreciation in a naturalistic condition with increased ecological value. Brain activation data revealed that broad emotion-related limbic and paralimbic regions as well as the reward circuitry were significantly more active for familiar relative to unfamiliar music. Smaller regions in the cingulate cortex and frontal lobe, including the motor cortex and Broca's area, were found to be more active in response to liked music when compared to disliked one. Hence, familiarity seems to be a crucial factor in making the listeners emotionally engaged with music, as revealed by fMRI data.

## Introduction

Listening to music is one of the most pleasurable human experiences, and one in which we invest a considerable amount of time and money. In a survey study [1], most subjects stated that their investment in this activity derives from the ability of music to convey emotions. For this reason, a better knowledge of how and why emotions are generated when listening to music will contribute to our understanding of why music is so important to our species.

With the present study, we investigated whether familiarity and aesthetic preferences in music have a role in determining the emotional involvement of the listener, and which of the two factors contributes the most to the recruitment of the limbic and reward centres of the brain. We aimed to do this by separating and individually analysing the role of these two factors in the enjoyment of music, therefore clarifying some of the questions raised by previous studies, in which one or both of these factors were not satisfactorily controlled.

Most studies investigating the psychological and neural basis for the impact of music on our emotions have focused on perception, induction, and recognition of basic emotions, such as happiness and sadness. For instance, converging evidence shows that acoustic features such as melody and tempo are relevant in determining the happy and sad emotional connotations of music (see, for example, [2], [3]). Happy music is usually characterized by fast tempo and major mode, while sadness in music is expressed by slow tempo and minor mode [2], [4], [5]. It has been proposed that basic emotions are the immediate affective responses to music, likely mainly originating from the brainstem, which derive from the association or mimicking of acoustic cues present in the music with those residing in emotional (human or animal) vocalizations [6]-[9].

A slower emotional response is musical enjoyment, which refers to an aesthetic emotion originating from the appraisal of the acoustic and formal properties of the music. Enjoyment is strongly modulated by individual factors, such as familiarity with the music, personality, current mood, and taste [8], [9]. The aesthetic emotion of enjoyment leads to conscious judgements of liking, i.e., the positive or negative judgement about a musical piece, and hence, the degree of enjoyment can be measured by liking ratings. In some rare occasions in which musical enjoyment is particularly strong and intense, physiological responses, namely frissons (including chills and goose bumps; for a review, see [10]), also occur. Frissons can be measured with the polygraph. Those body changes, however, cannot be considered as the sole measure of musical enjoyment as they are triggered in only a small percentage of subjects (mainly musicians), typically with familiar music and in correspondence of abrupt harmonic or timbral variations, hence not just when listening to any favourite musical piece [10], [11].

Capitalizing on the established theoretical model of basic emotions developed in the visual domain [12], perception, recognition and induction of basic emotions in music have been repeatedly studied, e.g., with questionnaires [13], by testing brain-lesioned patients [5], by recording autonomic nervous system reactions [14], [15], and by measuring central nervous system responses [16], [17]. In contrast, very little is known about music enjoyment, and the research regarding pleasurable feelings derived from music has been largely confined to studying neural and physiological correlates of chills, and contrasts between consonant and dissonant wrongly-sounding music [18]-[21]. Music-induced chills and consonant music activated brain areas known to be involved in reward and positive emotions, such as the nucleus accumbens (NAc), the ventral tegmental area (VTA), the orbitofrontal cortex, and the ventromedial prefrontal cortex [18], [21]. In turn, the subjective decision of consciously liking a piece of music, and the related joyful, pleasurable feelings associated with it, have only started to be explored by our group (for a review, cf. [22]), also prompted by the powerful effects of exposure to favourite pop/rock songs on cognitive recovery and mood improvement after middle cerebral artery stroke [23]. For instance, brain regions previously associated with affective processing and evaluative judgements, such as the insula, the anterior cingulate cortex, and the ventromedial prefrontal cortex, were associated with conscious liking of music, whereas recognition of happy or sad emotional connotations in music activated mainly auditory regions and the insula (Brattico et al., in preparation).

An important individual factor determining the variation of musical enjoyment and liking, as well as the occurrence of frissons in response to music, is familiarity: becoming more familiar with a particular piece of music increases the subject's liking ratings for it [5], [24], [25]. This phenomenon, known in the literature as the *mere exposure effect*, suggests that familiarity might play an important role in the emotional engagement of listeners with the music. The neural mechanisms governing this *mere exposure effect* are, however, still unrevealed. Furthermore, several imaging experiments looking for brain activations to familiar/unfamiliar music have been performed, but the use of different techniques, stimuli and tasks have yielded somewhat different results. Using positron emission tomography (PET), Satoh and collaborators [26] reported activations in the anterior portion of bilateral temporal lobes, posterior portion of superior temporal gyri, anterior and posterior portion of medial frontal lobes, bilateral cingulate gyri, left inferior frontal gyrus (IFG) and middle portion of the left superior temporal gyrus (STG). The described regions were obtained by subtracting a familiarity task (judging whether melodies were familiar or not) and an alteration-detection task (detecting altered notes), in a set of melodies played with a synthesizer. In a functional magnetic resonance imaging (fMRI) study [27], the neural basis of familiarity was analysed using classical music excerpts and odours, showing activations for familiar over unfamiliar music in left frontal regions, namely in the superior frontal gyrus (SFG), medial frontal gyrus (MFG) and precentral gyrus (pCG), and also in the left superior temporal sulcus (STS) and parietal regions, such as the posterior part of the left cingulate gyrus, the right angular gyrus (AG) and the left supramarginal gyrus. Additionally, the authors described a vast network of overlapping left hemisphere activations for familiar over unfamiliar music and odours, including the SFG, IFG, AG, precuneus and parahippocampal gyrus, suggesting that there might be a multimodal neural system for the feeling of familiarity, which is independent of the sensory modality. Another recent fMRI study [28] showed that familiar monophonic melodies over acoustically balanced unfamiliar melodies (consisting in the reversed versions of the familiar ones) activated bilateral STS with a bias to the right, and that familiar music over random tones recruited the right supplementary motor area (SMA), the planum temporale and the IFG. Interestingly, in both these contrasts, the authors observed sub-threshold activations in the ventral striatum and precuneus. The ventral striatum activation is of particular interest to our study since it includes the NAc, which receives projections from the dopaminergic neurons of the VTA and is therefore a central structure of the reward/pleasure system (cf. [29]). Although below threshold, this activation is consistent with our hypothesis that familiarity is an important factor for the emotional engagement and/or induction of pleasurable feelings in the listener.

Music fruition is a highly subjective experience, which varies widely across individuals. While listening to music, we can be moved by the melody, or we may find ourselves focusing on a timbre of an instrument or combination of instruments, or else we can be emotionally engaged by abrupt changes in the harmony or rhythm. Hence, in order to mimic the naturalistic situation in which music appreciation occurs, we discarded the manipulation of a single music dimension, and rather used expressive music from the pop/rock music genre, as it is the most ubiquitous in Western world (and also very diffuse in non-Western populations; for a similar approach in neuroimaging research, see [30]). In addition, appreciation of pop/rock music does not require formal musical training, and it is consequently the most available and important instance of aesthetic enjoyment of music. In order to further enhance the experience of musical enjoyment and of familiarity with music, subjects performed a listening test prior to the fMRI measurements, from which a unique set of musical stimuli per participant was chosen.

The naturalistic approach adopted here has been used before us by Janata [30]. In that study, fMRI and pop/rock music that extended to subjects' childhood time to evoke autobiographical memories were used. The analyses of the parametric variation (the areas of activation for the independent effects) of familiarity, autobiographical salience and valence showed that the most widespread activations were observed for the familiarity regressor. These activations included the IFG, medial frontal gyrus (MFG), pre-SMA, medial prefrontal cortex, STG, AG, medial temporal gyrus (MTG), cerebellum, caudate nucleus and ventro-lateral thalamic nucleus. A series of cortical and subcortical activations correlated with the degree of experienced positive affect were also reported, namely in the left superior frontal sulcus (SFS), right STG, left ventral anterior cingulate cortex, left substantia nigra and left ventral lateral thalamic nucleus. That study [30], however, focused on testing if the medial prefrontal cortex has a role in the association of musical features with autobiographical memories and emotions, rather than studying the brain areas recruited by familiarity and liking of music. For instance, the ratings of valence and pleasantness do not allow to tackle the subjective liking of music, since they might be driven by acoustic features and sensory processing. Moreover, since the participants classified each song during the fMRI recordings, the number of stimuli obtained for each condition was different, and had, therefore, different statistical weights in the final model.

In sum, with this study, we examined the role of familiarity and aesthetic preferences in music enjoyment and in the activation of limbic and reward centres in the brain, using commercially available pop/rock songs. In an initial phase, candidates participated in a listening test, in which they listened to song extracts and decided if each song was familiar or unfamiliar and if they liked it or not. Based on this test, a unique set of stimuli to be presented during an fMRI session was selected for each participant, containing music in four different conditions: familiar liked, familiar disliked, unfamiliar liked and unfamiliar disliked. With this procedure, we were able to obtain the same number of stimuli for each condition, which, in turn, allowed us to determine the brain structures associated with familiarity and liking of music. Based on previous literature on the *mere exposure effect* [5], [24], [25], we expected to find that familiarity has an important role in the pleasurable emotions derived from music listening. In particular, we expected that familiar songs would elicit strong activations in limbic and reward system regions of the brain.

## Methods

### Ethics Statement

This study used healthy human subjects as listeners in an fMRI experiment. All the participants were previously informed of the conditions of the study and gave written informed consent. The experiment was conducted according to the ethical guidelines of the Declaration of Helsinki and was approved by the Institutional Review Board of the Ginoeco Clinic, where the experiment was performed.

### Participants

Twenty-seven volunteers participated in the first phase of the study, i.e., the listening test, but only fifteen gathered all the conditions to undergo the second phase, i.e., the fMRI experiment. One of these participants was excluded from the results due to excessive movement in the scanner. Fourteen right-handed adult subjects (9 males; ages 24-40, mean 32), without known auditory impairments, neurological diseases, ferrous implants or claustrophobia, participated in the fMRI experiment. None of them was a professional musician nor had taken formal musical lessons in the recent years, and all of them considered themselves as music lovers.

### Listening test

Subjects who reported being “music lovers”, and actively listened to music everyday but had minimal (and distant in time) formal musical education, were invited to participate in a listening test prior to the imaging experiment. During this test, they heard 15 sec excerpts of 110 pop/rock songs from several decades, all available on commercial CD's (please check table 1 for the list of songs). The song extracts had 5 sec of silence between them, allowing the subjects to answer two questions for each song. The first question was: is this song familiar or unfamiliar to you? They were instructed to choose “familiar” when they were certain to know the song and could anticipate what comes next; in contrast when they did not know the song at all or think they might have heard it before but were not sure, participants were instructed to answer “not familiar”. They also had to classify each song extract in terms of liking or disliking, using a Likert scale from 1 to 10, and had a graphical representation to help visualize the scale.

**Table 1:** List of song extracts presented during the listening test.

Supertram - *Right*	Soap & Skin - *Fall Foliage*	Portishead - *Glory Box*	Starship - *We Built This City*	Zita Swoon - *Thinking About You All the Time*
Cat Power - *The Greatest*	Alicia Keys - *No One*	Devendra Banhart - *Dogs they Make Up the Dark*	Rufus Wainwright - *What Can I Do*	The bravery - *The Ring Song*
Sabrina - *Boys*	Leona Lewis - *Better in Time*	Daniel Powter - *Bad Day*	Dj Assad vs. Maradja - *Summer Lovin*'	Portishead - *Machine Gun*
Interpol - *No I in Threesome*	The Sound - *Winning*	Led Zeppelin - *Immigrant Song*	The Books - *Be Good To Them Always*	The Acorn - *Hold Your Breath*
Queen of Japan - *I Love Rock'n Roll*	Taio Cruz - *She's Like a Star*	The Smiths - *Girlfriend in a Coma*	Marissa Nadler - *Thinking of You*	Heart - *Alone*
Cocorosie - *Beautiful Boyz*	Echo and the Bunnyman - *The Killing Moon*	Bon Jovi - *Make a Memory*	Sonic Youth - *Turquoise Boy*	The Castanets - *Glory B*
This Mortal Coil - *Another Day*	Gus Gus - *Remembrance*	Camera Obscura - *Don't Do Crowds*	George Michael - *Kissing a Fool*	Chop Wood - *Fiction In Disguise*
Le Freak - *Chic*	Pulp - *Common People*	Joy Division - *Transmission*	This Mortal Coil - *Sixteen Days Gathering Dust*	Laura Barret - *Robot Ponies*
Queen - *Friends Will Be Friends*	Air - *Once Upon a Time*	Beyoncé & Shakira - *Beautiful Liar*	Cleer - *So Good*	The Middle East - *The Darkest Side*
Chop Wood - *She*	Chop Wood - *Loud Statics*	Cat Power - *Metal Heart*	Taylor Dayne - *Tell It to My Heart*	Shaggy - *Feel the Rush*
Patrick Wolf - *Wind in The Wires*	Sting - *If I Ever Loose My Faith in You*	Chris Brown - *Take You Down*	Tortoise + Bonnie Prince Billy - *Love Is Love*	PJ Harvey + Thom Yorke- *This Mess We're In*
Marillion - *The Web*	Thom Yorke - *And It Rained All Night*	Bauhaus - *In the Flat Field*	Yann Tiersen - *Les Jours Tristes*	Gentlemen - *Intoxication*
Jeff Buckley - *Lover, You Should've Come Over*	Jeremy Warmsley - *Dancing With The Enemy*	Tindersticks - *Tiny Tears*	Patrick Wolf - *To the Lighthouse*	Bonnie Prince Billy - *No Bad News*
Joanna Newsom - *Only Skin*	Genesis - *I know What I Like*	Beirut - *The Penalty*	Yo la Tengo - *The Race Is On Again*	Tina Turner - *Typical Male*
Heidy Happy - *Hush*	Vincent Gallo - *Honey Bunny*	Belinda Carlisle - *Heaven Is a Place on Earth*	Phil Collins - *You Can't Hurry Love*	Jeremy Warmsley - *Temptation*
The Castanets - *Shadow Valley*	This Mortal Coil - *Song to the Siren*	Jeremy Warmsley - *Lose My Cool*	Cat Power - *Satisfaction*	Waiting For Eve - *La Route*
Ne-Yo - *Closer*	She Wants Revenge - *These Things*	Robert Palmer - *Addicted to Love*	Cindy Lauper - *True Colours*	Heidi Happy - *Push the Door*
Jay Sean - *Ride It*	Jordin Sparks - *One Step at a Time*	New Order - *Blue Monday*	Elliot Smith - *Son of Sam*	Kyte - *Bridges In the Sky*
Sonic Youth - *Incinerate*	Belle and Sebastian - *Dog on Wheels*	Antony and The Johnsons- *Man is the Baby*	Felt - *Riding on The Equator*	Architecture In Helsinki - *Souvenirs*
Bon Jovi - *Livin' On a Prayer*	Kat DeLuna - *Run the Show*	Chris Brown - *With You*	Laurie Anderson - *From The Air*	Supertramp - *Dreamer*
Arcade Fire - *No Cars Go*	Bonnie Prince Billy - *Strange Form of Life*	Architecture in Helsinki - *Scissor Paper Rock*	50 Cent + Justin Timberlake - *Ayo Technology*	Emiliana Torrini - *Birds*
*Madonna -* Live to Tell	Radiohead - *Karma Police*	F.R. David - *Words*	Rihanna - *Take A Bow*	Soap & Skin - *Spiracle*

Only participants who selected at least twelve songs in the conditions we wished to test (familiar and liked (FL), familiar disliked (FD), unfamiliar liked (UL) and unfamiliar disliked (UD)) were chosen to participate further in the experiment. For each participant, we chose the songs classified in the most extreme positions of the preference scale as possible, and the ones in the central part of the scale were ignored.

A minimum of two weeks passed between the listening test and the fMRI experiment, to avoid recognition of the songs classified as “unfamiliar”. After the fMRI experiment, subjects were asked if they recalled recognizing any song from the questionnaire that they had not heard before, and the answer was negative in all the cases.

### Stimuli and procedure of fMRI experiment

The stimuli consisted of 48 pop/rock songs, all with an instrumental and a vocal component (sang in English), twelve in each experimental condition (FL, FD, UL, UD). Although all participants are relatively fluent in English, it is not their maternal/primary language. The song extracts were digitized at a sampling rate of 44100 Hz, 32 bit, stereo. The baseline consisted of Morse code (MC), perceived by our sample of subjects (unfamiliar to the code) as a series of meaningless beeps. Stimuli were presented via noise cancelling headphones, and volume was adjusted to a comfortable level for each subject.

**Figure 1:**
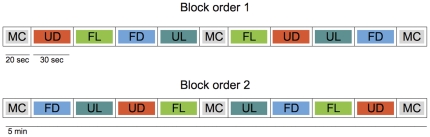
Sequence of blocks from the fMRI experiment. Graphical representation of the block sequence presented during the fMRI scans; baseline (MC) blocks had a duration of 20 sec while the remaining blocks lasted for 30 sec; total time for each run was 5 min and each participant had 6 runs (total time per participant = 30 min). MC: morse code; FL: familiar liked songs; FD: familiar disliked songs; UL: unfamiliar liked songs; UD: unfamiliar disliked songs.

In the fMRI experiment, a block design was chosen. Half of the participants were presented with the following block order for each of the six runs: MC UD FL FD UL MC FL UD UL FD MC; the other half heard the following (inverse) block order: MC FD UL UD FL MC UL FD FL UD MC (see figure 1). Each subject underwent the structural scan first, followed by six functional runs, lasting 5 min each. During each run, a total of 1 min was presented for each condition, in two 30-sec blocks. The baseline tones (MC) were presented in three 20-sec blocks, one at the beginning, one in the middle and one at the end of each run. Subjects were instructed to try to relax as much as possible and pay attention to the music without performing any explicit task. They were told to listen to the music and try to enjoy it (or not) as naturally as possible.

### fMRI acquisition and analysis

Images were acquired using a 1.5 Tesla Philips Gyroscan Intera whole-body MRI system (Philips Medical Systems, Best, The Netherlands) at the Ginoeco Clinic in Porto, Portugal. Changes in blood-oxygenation level-dependent (BOLD) signal were measured by using gradient-echo echo-planar-imaging (GE- EPI) with TR = 3000 ms, TE = 50 ms, and 90^°^ flip angle. The whole brain was covered with a total of 30 axial slices, with 4 mm thickness, 230×230 mm^2^ field of view, and a 64×64 acquisition matrix, yielding a voxel size of 3.5×3.5×4.0 mm^3^. A spoiled gradient recalled echo (SPGR) pulse sequence was used to collect high-resolution T1 -weighted structural images in the same session, with 1 mm thick axial slices of 230×230 mm^2^ field of view and a 256×256 acquisition matrix, yielding a reconstructed voxel size of 1 mm^3^.

FMRI data processing was carried out using FEAT (FMRI Expert Analysis Tool) Version 5.98, part of FSL (FMRIB's Software Library, www.fmrib.ox.ac.uk/fsl). The following pre-statistics processing was applied: motion correction using MCFLIRT [31]; non-brain removal using BET [32]; spatial smoothing using a Gaussian kernel of FWHM 5 mm; grand-mean intensity normalisation of the entire 4D dataset by a single multiplicative factor; highpass temporal filtering (Gaussian-weighted least-squares straight line fitting, with sigma = 100.0 s). Registration of the functional images to high resolution structural and standard space images was carried out using FLIRT [31], [33].

Statistical analysis of the images was accomplished in three levels. In the first level, each of the six runs of each participant was individually analysed. Time-series statistical analysis was carried out using FILM with local autocorrelation correction [34] using a GLM approach. Each condition was entered as an EV and contrasted to the other conditions and the baseline. In the second level analysis, the six runs of each participant were entered into a fixed effects model by forcing the random effects variance to zero in FLAME (FMRIB's Local Analysis of Mixed Effects) [35]-[37]. Several third level group analysis were carried out, one for each desired contrast, using FLAME stage 1 [35]-[37].

## Results

### Listening test data

**Figure 2:**
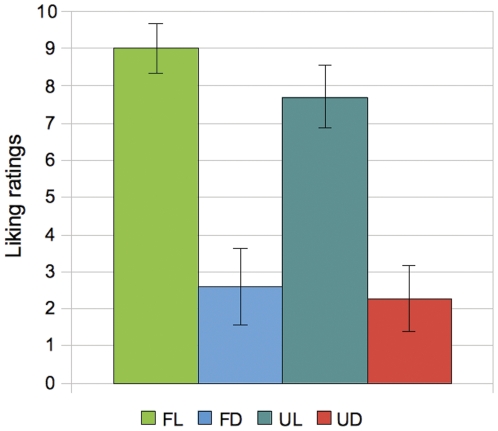
Listening test results. Medium liking ratings and standard deviation (14 subjects), per experimental condition, for the songs used in the fMRI experiment. FL: familiar liked songs; FD: familiar disliked songs; UL: unfamiliar liked songs; UD: unfamiliar disliked songs.

The analysis of the data from the listening test evidenced that, within the universe of songs selected for the fMRI experiment, the liking ratings for familiar songs were higher than for unfamiliar songs, both in the liked and disliked conditions (figure 2). In a scale of 1 to 10, the mean rating for the familiar liked songs was 9.01, while for unfamiliar liked ones was 7.7. Disliked though familiar songs had a mean rating of 2.57, while disliked and unfamiliar songs achieved only 2.26.

### fMRI data

**Figure 3:**
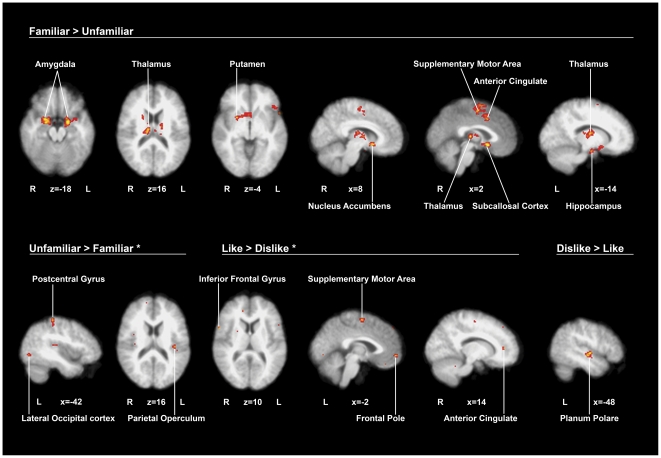
fMRI results. Statistical maps superimposed on standard brain in the MNI space. Images were thresholded using clusters determined by Z>2.5 and a corrected cluster significance threshold of P = 0.05. When marked with asterisk, images were thresholded at P = 0.005 uncorrected. Coordinates are presented in mm. L =  left hemisphere; R =  right hemisphere.

As a general result of listening to music, several significant activations were observed both in cortical (mainly temporal and frontal) and subcortical (limbic, paralimbic and reward system) regions. We further explored the contribution of familiarity and musical preferences to this general pattern of brain activation. Activated regions for each contrast are described bellow, and details can be found in Table 2. Main activations for the four contrasts can be visualized in figure 3.

**Table 2:** List of significant activations.

Contrast	Anatomical regions	Z	x	y	z
Familiar > Unfamiliar	Left amygdala	3.91	-20	-2	-18
	Left temporal pole	3.89	-54	16	-10
	Right SMA	3.87	4	-6	56
	Right subcallosal anterior cingulate cortex	3.85	2	14	-8
	Right amygdala	3.76	22	-2	-18
	Left SMA	3.75	0	0	66
	Right thalamus	3.66	12	-16	16
	Left thalamus	3.65	-10	-6	10
	Left subcallosal anterior cingulate cortex	3.65	-2	14	-8
	Left putamen	3.60	-16	8	-12
	Right putamen	3.48	26	8	-4
	Right dorsal anterior cingulate cortex	3.41	2	12	40
	Left hippocampus	3.41	-14	-8	-20
	Left frontal orbital cortex	3.39	-44	22	-10
	Left paracingulate gyrus	3.25	0	8	48
	Right accumbens	3.21	8	10	-8
Unfamiliar > Familiar*	Left postcentral gyrus	3.76	-38	-32	62
	Left parietal operculum / insula	3.24	-38	-26	16
	Left lateral occipital cortex, inf. div.	3.17	-42	-78	-4
	Right occipital pole	3.16	16	-92	20
Like > Dislike*	Right SMA	3.38	6	-6	60
	Right rostral anterior cingulate cortex	3.22	14	44	8
	Left frontal pole	3.20	-2	58	-6
	Left SMA	3.17	-2	-8	62
	Left MFG	3.12	-28	20	48
	Right IFG, pars opercularis	3.08	58	10	10
	Left IFG	3.03	-52	18	14
	Left SFG	3.02	-22	28	50
Dislike > Like	Left planum polare / STG	3.64	-48	-12	-2
	Left STG, post div	3.60	-60	-22	2

Statistic images were thresholded using clusters determined by Z>2.5 and a corrected cluster significance threshold of P = 0.05. When marked with asterisk, statistic images were thresholded at P = 0.005 uncorrected. Coordinates are in the MNI space and presented in mm.

### Music *>* Baseline

In the music (irrespective of whether it is familiar or not and liked or not) vs. baseline condition, extensive activations were observed bilaterally along the STG and SFG. In the left hemisphere, activations were also found in the supramarginal gyrus and planum temporale, extending more posteriorly than on the right hemisphere. Also, the supplementary motor cortex showed bilateral activations. In addition, a series of activations were also observed in structures from the limbic and reward systems, namely in the amygdala, nucleus accumbens, caudate, anterior cingulate cortex, hippocampus and parahippocampal gyrus.

### Familiar music *>* Unfamiliar music

When contrasting familiar (FL + FD) with unfamiliar (UL + UD) songs, several clusters of significant activations emerged (corrected, Z>2.5, P = 0.05). Activated areas include the anterior cingulate cortex (including dorsal and subcallosal parts), amygdala, thalamus and putamen bilaterally. Also the right nucleus accumbens showed increased activity for familiar over unfamiliar music. Another cluster comprises the supplementary motor cortex bilaterally, the dorsal part of the right anterior cingulate cortex and the left paracingulate. Several other regions were also active in the left hemisphere, particularly the hippocampus, the temporal pole and the frontal orbital cortex.

### Unfamiliar music *>* Familiar music

When adopting the threshold for statistical significance corrected for multiple comparisons, we did not observe any significant activation for the contrast unfamiliar music > familiar music. However, since this null finding is not physiologically viable, we conducted the analysis with an uncorrected threshold of P = 0.005 in order to explore activations for this contrast and compare them with the familiarity contrast. In this analysis, we found that unfamiliar (UL + UD) over familiar (FL + FD) music yielded several active regions in the left hemisphere, which included the postcentral gyrus, the left parietal operculum cortex including Heschl's gyrus and the insula, and the inferior division of the lateral occipital cortex. We also observed a small cluster in the right occipital pole.

### Liked music > Disliked music

Also, the contrast liked music > disliked music did not yield any significant activation when using corrected threshold. Hence, we conducted again the analysis with an uncorrected threshold of P = 0.005. In this analysis, we found that liked music contrasted with disliked music activated bilaterally the supplementary motor cortex. On the right hemisphere, the pars opercularis of the IFG and the rostral anterior cingulate cortex also showed increased activation. In the left hemisphere, significant activations were more extensive than in the right hemisphere, and include the SFG, MFG, IFG and frontal pole.

### Disliked music > Liked music

The contrast between disliked songs (FD + UD) and liked songs (FL + UL) produced one cluster in the left hemisphere (corrected, Z>2.5, P = 0.05), which included activations in the planum polare and STG (posterior division).

## Discussion

In this study, we used pop/rock songs that people listen to in everyday life [38] to investigate how musical preferences and familiarity modulate the activity of brain regions recruited during music listening and appreciation. We found that musical preferences had only a marginal effect on the activation of limbic, paralimbic and reward system areas. On the contrary, familiarity with the music was the key factor to trigger increased blood oxygen level dependence (BOLD) response in these emotion-related regions, namely in the putamen, amygdala, nucleus accumbens, anterior cingulate cortex and thalamus.

Emotional responses to music have previously been shown to recruit limbic, paralimbic and reward structures of the brain. However, it was not clear how factors such as familiarity and musical preferences interact in modulating activity in these brain regions. In our study, we found that most emotion-related brain activity was triggered by familiar (liked or disliked) music rather than liked (familiar or unfamiliar) music, thus supporting our hypothesis about the crucial role of the familiarity factor in music appreciation and induction of emotions in the brain.

Blood and Zatorre [20] have reported a correlation between increased intensity of felt chills when listening to favourite pieces of music and activations or deactivations of such regions, namely the nucleus accumbens in the ventral striatum, midbrain, amygdala, orbitofrontal cortex and ventral medial prefrontal cortex. Although it was not emphasized, the pieces of music used were highly familiar to the participants, since they were given the chance to choose the ones that consistently elicited intense pleasure and chills. In turn, Brown and collaborators [39] used unfamiliar though pleasurable music, and described activations in the ventral anterior cingulate cortex, the hippocampus, anterior insula and also the nucleus accumbens. It is worth noting, though, that the activation they reported in the nucleus accumbens is sub-threshold. In our study, instead, no neural activity in the ventral striatum was obtained in response to liked music, even after using a more liberal statistical threshold without correction. One possible reason for the discrepancy with the results obtained between Browns' [39] and our study is that, although the exact time of the stimulus duration in their study is not specified, it was probably much more than the thirty seconds we used. Moreover, they had only two functional scans, each one with a different song (probably the entire song), allowing the subjects to have more time to get emotionally engaged with the unfamiliar song. It may then be hypothesized that a longer exposure to unfamiliar (and liked) music in our study would have generated stronger responses in the limbic system too. In a series of studies [40], [41], however, it was found that musical excerpts of 1 sec only were enough to allow the recognition of basic emotions of happiness and sadness in the participants, and that this effect is weakly influenced by musical expertise and excerpt duration. Another experiment [42] showed that the time that participants required to initiate an emotional judgement is shorter for familiar than for unfamiliar music, which may indicate that also the emotional involvement (i.e. the feeling of emotions, which is different from the identification of emotions) can be modulated by familiarity. Nevertheless, the time course of emotional responses during music listening has not been investigated in neuroimaging studies, and hence, should be the focus of future investigations.

The two studies addressing the pleasurable feelings derived from music that we discussed so far have used PET, and it is possible that this technique lacks the resolution to accurately locate small structures like the nucleus accumbens. A more recent study [19] used fMRI and functional and effective connectivity to show that listening to music has a strong effect in mesolimbic structures of the reward circuitry like the nucleus accumbens and the ventral tegmental area, but also in the hypothalamus and the insula. Another very recent paper [29] clearly shows the release of dopamine in the mesolimbic reward system in correlation with intense pleasurable experiences elicited by music. Even more interesting, it shows that, in anticipation of these peak emotional responses, the caudate nucleus was more active, while during the experience of the peaks themselves, increased activity was found in the right nucleus accumbens. It happens that, in this study, the authors used music that was highly familiar to the participants, but did not satisfactorily control for the familiarity of the neutral musical stimuli, leaving open the possibility that this factor might have contributed to the described activations. Moreover, only 8 out of about 200 subjects showed a consistent peak emotional response to music and were thus selected for the study, making it difficult to generalize these results to the overall population. In our study, we also found increased BOLD response in the right nucleus accumbens, curiously with the local maxima in the same coordinates as in Salimpoor et al. [29], but only for familiar music. This means that, in previous studies where familiarity was not properly controlled, the activations of this brain structure might have been wrongly attributed to the sole feeling of liking, discarding the crucial role of familiarity.

We also obtained, with familiar songs, strong bilateral activations in the amygdala and the subcallosal cingulate cortex. Both these regions have been previously correlated with the emotional responses to musical stimuli. Amygdala activations were associated with sad music [17], unpleasant music [18] and both familiar and unfamiliar music [43]. The subcallosal part of the anterior cingulate cortex has also been shown to be active, especially with pleasant/consonant music (see [18], [21], [39]).

Also the putamen showed bilateral increased activity for familiar music, which can be accounted for the motor synchronization to the rhythm of the pop/rock excerpts; the same function can also be attributed to the activations observed in the thalamus (for a review, see [44]). Similar to our results, Brown and collaborators [45] further showed the recruitment of the putamen, with emphasis on the right side, while subjects were watching dancers moving to a regular, metric rhythm. Of course, people can synchronize to rhythm and dance to unfamiliar music as well, but possibly the activation of the basal ganglia structures might indicate that familiarity with the musical stimulus is a prominent factor in engaging the listeners also motorically, besides emotionally.

Another cluster of activation for familiar over unfamiliar music was located in the SMA. Our interpretation of these activations is that the participants might have silently sung the familiar tunes. This is consistent with the proposal by Halpern and Zatorre [46] and Halpern [47] that this particular region is activated during musical imagery, or the act of imaging music in our minds, something that is likely to happen when you know a song and can predict what comes next.

Several behavioural studies (namely [5]) confirmed what has been previously described by Meyer [24], which is the positive effect of prior exposure on music liking, also called the *mere exposure effect.* These results were also reproduced in our listening test, where we observed that within the group of songs that fitted the aesthetic preferences of each participant, the ones that were familiar were the most highly rated in terms of liking (figure 2). Accordingly, the brain results showed that familiar songs, including those that were liked and those that were disliked, were efficient in activating the network of brain regions known to respond to emotional stimuli. Another experiment [25] reported an effect similar to the mere exposure effect, this time also considering the valence (happy and sad) of the musical stimuli and the quality of the listening method (focused or incidental). They found that the effects of exposure on liking are different for focused and incidental listening, namely that liking ratings were higher for happy songs, but only in the focused listening condition. They also observed that liking ratings increased linearly as a consequence of exposure, but only in the incidental listening condition. In the focused listening condition, liking ratings were represented by an inverted U in function of exposure, meaning that the repeated exposure initially increases the ratings of the songs, but it then tends to cause an “over-familiarity” effect, reflected in the decreasing of the ratings. These studies suggest that the mere repetition of melodies is sufficient to increase the affective responses to these melodies, at least in an initial stage.

To our knowledge, we provide the first functional neuroanatomical evidence for a strong effect of familiarity in the way listeners' get emotional engaged with the music, at least within an experimental setting. Our results not only strengthen the body of evidence showing that music is very efficient in recruiting emotional centres of the brain, but also clearly provide evidence that familiarity with a particular piece of music is an extremely important factor for emotional engagement, and thus furnishes “direct access” to these emotional centres of the brain.

We would also expect that, besides familiarity, musical preferences would also be an important factor to determine the emotional involvement of listeners, but, in our study, the aesthetical preferences of the participants generated only focal activations in brain regions, including limbic ones. In particular, liked songs (compared with disliked ones) activated the supplementary motor cortex, the right anterior cingulate cortex and a network of frontal regions. The anterior cingulate has been implicated in aesthetic judgement processes by studies in the visual domain [48], [49]. In Kawabata's paper [48], the anterior cingulate was recruited when the subjects viewed and judged beautiful stimuli (in contrast to neutral stimuli), which is consistent with the activation we found in this structure for liked, more than disliked, music. Also the inferior frontal gyrus, another region that we found to be active in this condition, was implicated in the aesthetic judgements of beauty with visual stimuli (see [50]). Furthermore, the activation on the frontal pole/frontal medial cortex is also consistent with studies of the neural basis of evaluative judgements, namely [51], [52]. It seems likely, then, that although participants were instructed to just listen to the music and not to perform any active task, involuntary aesthetic judgements happened, and reflected subject's positive appreciation of the songs presented in this condition. Despite the fact that, in this experiment, we obtained only few activations in limbic regions and absence of activity in the reward system regions for liked music (more than disliked one), we know from our own private experiences that listening to a loved song is drastically different from listening to a disliked one. Nonetheless, when listening to only thirty second extracts of songs inside an MRI machine, the effect of the aesthetical preferences most likely gets diminished, and familiar songs have an advantage in emotionally engaging the listener. A more risky explanation for this result might be the assumption that one thing is our aesthetical taste, and another thing is what we are hardwired to like, which may be even difficult to admit in public for social reasons. In other words, subjects could have classified part of the songs based on their aesthetical construct and not on the “real” feelings elicited by the music. A recent study [53] showed that, in adolescents, song popularity had a significant effect on the participants' likeability ratings of the songs, showing that conscious knowledge of the song popularity may influence people to switch their choices towards the consensus. In that study [53] it was further suggested that such a switch might occur to minimize the anxiety generated by the mismatch between individual and group preferences. Such findings raise the question whether similar social constraints come into play every time an individual is asked to make an aesthetical judgement, including in the laboratory setting.

In the opposite contrast, disliked songs versus liked ones, we obtained no evidence of brain activations related to affective or cognitive processing, since only auditory-cortex regions were active, namely the left planum polare and STG. These regions are known to be recruited for perceptual integration of sound features into auditory objects, timbral processing, and musical scale rule extraction [54]-[57].

The regions found to be more active for unfamiliar songs rather than familiar ones included rolandic/parietal operculum areas, as well as occipital cortex areas. Plailly and collaborators [27] have previously reported two clusters in the vicinity of ours, namely the ones in the left postcentral gyrus and left parietal operculum, for unfamiliar minus familiar music. We think these activations may be related either with the attempt to recall the songs or to the detection of novelty, although the latter action has been described to activate more medial parietal and temporal lobe regions (see, for example, [58]). Nonetheless, it is worth noting that the activations for unfamiliar versus familiar songs were observed only after a more liberal threshold was applied, and that this overall pattern of activation is significantly more reduced than that of the opposite contrast.

It is worth noting that our results are consistent with the role proposed by Rauschecker and Scott [59] for the dorsal stream of their dual stream model. A further development on the role of this dorsal auditory pathway was recently accomplished by Rauschecker [60]. This model postulates that when incoming sounds match expectations based on previous learning, the premotor cortex and basal ganglia are recruited. Parietal cortex regions may have a special role in comparing the incoming sounds with those expectations and they most likely are activated when the expectations are not matched, what happens when the sounds are unfamiliar. In our data, we found activations of the supplementary motor cortex and putamen (basal ganglia) for familiar sounds and of parietal rolandic operculum for unfamiliar sounds, thus supporting the role of this dorsal stream for processing sensorimotor sound events and matching (or unmatching) them with learned ones.

Finally, our results also show that it is possible to use complex acoustic stimuli in the form of commercially available music, and still find highly consistent activations across subjects, in contrast to the trend of using unexpressive, controlled stimuli, quite distant from the real music listened to in everyday life.
